# The effect of haloperidol’s perioperative application on postoperative delirium in elderly patients: a systematic review and meta-analysis

**DOI:** 10.1186/s12871-024-02434-8

**Published:** 2024-02-03

**Authors:** Meinv Liu, Jie Su, Bei Wang, Dongdong Yu, Jianli Li, Xinyu Cao

**Affiliations:** 1 Department of Anaesthesiology, Hebei General Hospital, Shijiazhuang, 050051 China; 2 Department of Gynaecology, Hebei General Hospital, Shijiazhuang, 050051 China; 3 Graduate School of Hebei Medical University, Shijiazhuang, Hebei Province 050011 China

**Keywords:** Postoperative delirium, Haloperidol, Meta-analysis, Perioperative period

## Abstract

**Objectives:**

To systematically review the evidence about the effect of haloperidol on postoperative delirium in elderly patients.

**Methods:**

PubMed, Embase, the Cochrane Library and China National Knowledge Infrastructure were used to find concerned studies for meta-analysis. The main outcome was the incidence of postoperative delirium, and the secondary outcomes were side effects of haloperidol and the length of hospital stay. The meta-analyses were conducted using the Review Manager Version 5.1. This study was conducted based on the PRISMA statement.

**Results:**

Eight RCTs (1569 patients) were included in the meta-analysis. There was a significant difference in the incidence of postoperative delirium between haloperidol and control groups (OR = 0.62, 95%CI 0.48–0.80, *P* = 0.0002, I^2^ = 20%). In addition, side effects of haloperidol and the duration of hospitalization were comparable (OR = 0.58, 95%CI 0.25–1.35, *P* = 0.21, I^2^ = 0%; MD =-0.01, 95%CI -0.16-0.15, *P* = 0.92, I^2^ = 28%). Subgroup analysis implied the effect of haloperidol on postoperative delirium might vary with the dose (5 mg daily: OR = 0.40, 95%CI 0.22–0.71, *P* = 0.002, I^2^ = 0%; <5 mg daily: OR = 0.72, 95%CI 0.42–1.23, *P* = 0.23, I^2^ = 0%).

**Conclusions:**

The meta-analysis revealed perioperative application of haloperidol could decrease the occurrence of postoperative delirium without obvious side effects in elderly people, and high-dose haloperidol (5 mg daily) possessed a greater positive effect.

**Supplementary Information:**

The online version contains supplementary material available at 10.1186/s12871-024-02434-8.

## Introduction

Postoperative delirium (POD) was one of the most prevalent central nervous system complications following general anaesthesia and surgery, particularly in elderly patients [1]. The incidence of POD ranged from 11 to 51%, with the highest prevalence in the patients undergoing cardiac and major non-cardiac surgeries [2]. It was described as an acute mental status shift characterized by inattention and changed degree of consciousness, which usually appeared within 5 days after the operation [3]. Considering the increasing number of elderly people undergoing surgery, POD required more research and attention due to the fact that POD could cause modest to severe physical impairments, including self-extubation, catheter displacement, long-term postoperative cognitive dysfunction and higher death risk [4]. Furthermore, it might lead to a decrease in the ability to live alone and affect the long-term quality of life, which brought enormous social and economic load. Consequently, appropriate intervention is necessary to decrease POD, especially in the aged.

The management strategies for POD included non-pharmacological measures and pharmacological measures [5]. Non-pharmaceutical interventions of POD contained staff education, early mobilization, pain control, reorientation, sleep-wake cycle preservation, and optimization of hydration and nutrition [5–8]. Non-pharmacological measures could provide a relaxing and soothing environment for patients, but it was challenging to implement the non-pharmacological measures due to clinical practice limits. Furthermore, many studies about non-pharmacological measures in preventing POD were poor quality and rather heterogeneous in design. As for pharmacological measures, dexmedetomidine, benzodiazepines, melatonin or ramelteon (an agonist of melatonin), and antipsychotic drugs were the most frequently employed to treat POD in clinical practice. Up to date, the mechanism of preventing POD with dexmedetomidine was unknown, it might be attributed to the protective effect of dexmedetomidine on ischemia-reperfusion injury [9]. However, the use of dexmedetomidine was restricted in some individuals owing to its adverse effects such as bradycardia and respiratory suppression. Moreover, researches on dexmedetomidine treatment during surgery to reduce POD were still controversial. As for benzodiazepines, available evidence suggested that it might enhance the likelihood and duration of delirium, particularly in the old [10]. Additionally, melatonin and its agonist ramelteon had the potential to reduce delirium incidence in ICU patients [11, 12], but a meta-analysis showed that the evidence was weak [13]. Hence, antipsychotic drugs might be a preferable choice for POD. Haloperidol, a typical butyrophenone-type antipsychotic, could block dopamine receptors in the brain, increase acetylcholine levels and regulate immune function [14, 15]. Moreover, haloperidol was regarded as a first-line treatment for POD due to the fact that it possessed anti-hallucinatory, anti-delusional and anti-agitation effects [16].

According to a study from Fukata et al., early prophylactic administration of haloperidol reduced the incidence of POD [17]. Teslyar et al. discovered that haloperidol not only possessed good effect on current delirium symptoms, but it decreased the occurrence and severity of delirium [18]. On the contrary, Hollinger’s study indicated that the use of haloperidol exerted no effect on POD improvement [19]. Besides, a previous study showed that the application of haloperidol could not alleviate postoperative neuroinflammation and cognitive impairment in aged rats [20].

In view of the controversy and gaining the latest evidence, this meta-analysis was conducted to evaluate the effectiveness of haloperidol on POD in elderly patients.

## Materials and methods

The meta-analysis adhered to the PRISMA guidelines, and a PRISMA checklist was provided in Supplementary Material 1.

### Search strategy and selection criteria

Two independent investigators thoroughly performed searches using PubMed, Embase, the Cochrane Library and China National Knowledge Infrastructure (CNKI) for RCTs concerning the administration of haloperidol to treat POD from the establishment of the database to April 26, 2023. Search strategy utilized a combination of medical subject headings (MeSH) words and free text words. The following search terms were used: (haloperidol OR haldol) AND (Postoperative Delirium OR Delirium, Emergence OR Emergence Agitation OR Agitation, Emergence OR Agitations, Emergence OR Post-Operative Delirium OR Delirium, Post-Operative OR Post Operative Delirium OR Postanesthetic Excitement OR Excitement, Postanesthetic OR Anesthesia Emergence Delirium OR Delirium, Anesthesia Emergence OR Emergence Delirium, Anesthesia OR Delirium, Postoperative OR Agitated Emergence OR Emergence, Agitated OR Emergence Excitement OR Excitement, Emergence).

**Fig. 1:**
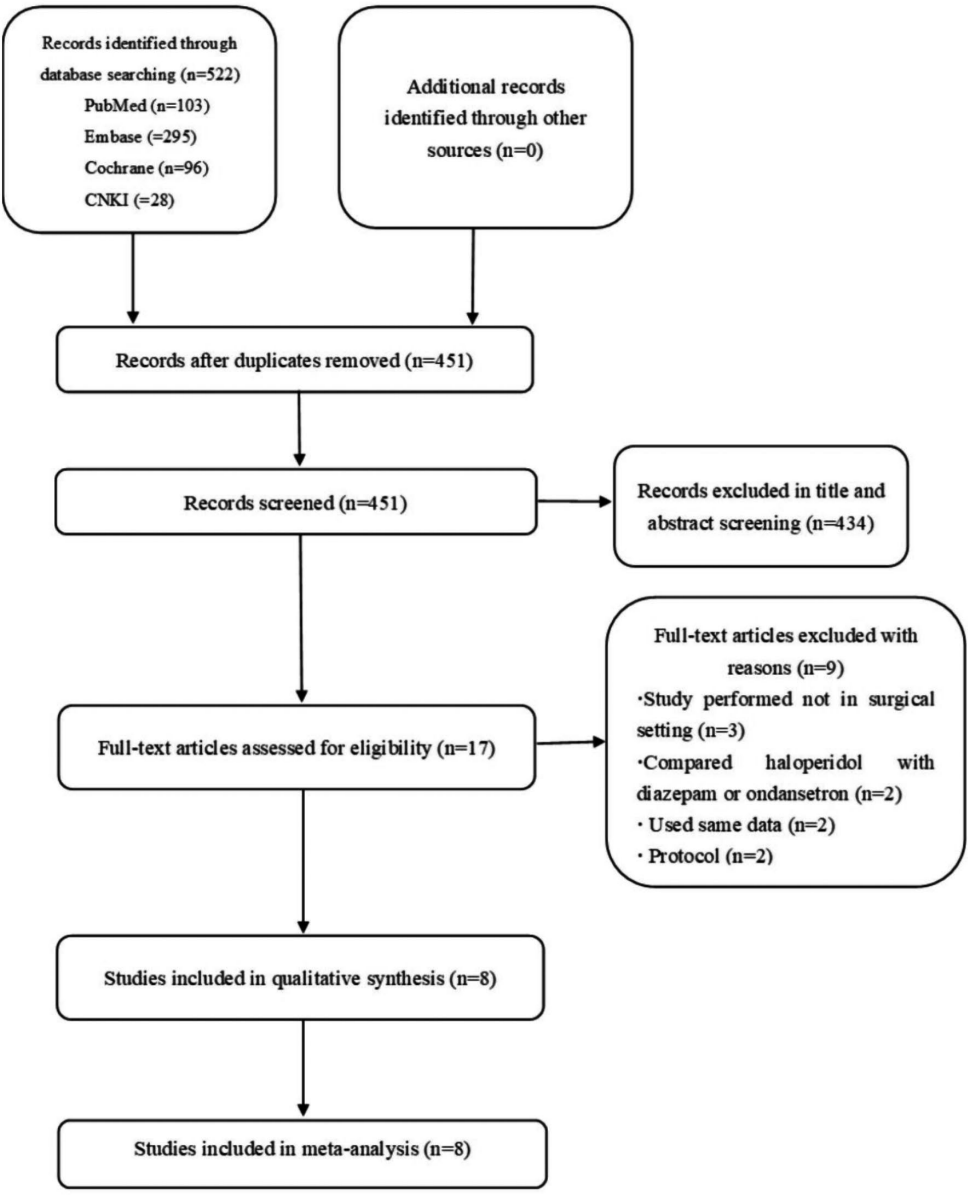
Flow diagram of the literature selection

Studies were included if they complied with the PICOS guideline: (1) Population: elderly patients undergoing surgery; (2) Intervention: only haloperidol; (3) Comparison: normal saline or no intervention; (4) Outcomes: the incidence of POD; (5) Study design: randomized controlled trials. These conditions were specifically excluded: (1) studies could not obtain full texts, case reports, conference abstracts and review papers; (2) patients were given haloperidol combined with other sedatives (dexmedetomidine or esketamine) in studies. There were no limits on language, administration timing or dosage of haloperidol. Two investigators assessed the titles and abstracts to ensure whether studies met eligibility and exclusion criteria, and then reviewed the full-text articles once reached a consensus. If necessary, a third reviewer was consulted to resolve any disagreements. The search flow chart was depicted in Fig. 1.

### Data extraction and quality assessment

All the corresponding information (first author’s name, publication year, country, range of age, number of participants, type of surgery, administrations for patients, occurrence of POD, side effects of haloperidol and duration of hospitalization) were extracted independently by two reviewers from each included study. We evaluated the risk of bias in enrolled studies from seven different parameters (Random sequence generation, Allocation concealment, Blinding of participants and personnel, Blinding of outcome assessment, Incomplete outcome data, Selective reporting and other bias) based on the Cochrane collaboration’s approach. The assessment of each indicator was divided into low risk, high risk, or unclear risk, which was presented in the risk of bias graph. Furthermore, the quality of evidence was assessed using the GRADE approach by the GRADEpro software. If there were contradictions in the procedures of information collection and literature quality assessment, a third assessor was consulted.

### Statistical analysis

Continuous data were analysed using the mean difference (MD) with a 95% confidence interval (CI) and dichotomous data using the odds ratio (OR) with 95% confidence interval. The heterogeneity was reflected by I^2^ statistics, and I^2^ < 50% implied the heterogeneity was small, homogeneous data were combined using a fixed effect model. In contrast, I^2^ > 50% showed that heterogeneity was substantial, and a random effects model was utilized to compute pooled effect size. A sensitivity analysis was conducted to determine whether removal of a single research would affect the entire findings of the meta-analysis. The publication bias was assessed by using funnel plots. Meta-analyses were conducted using the Review Manager Version 5.1 (The Cochrane Collaboration, Software Update, Oxford, UK). A *P* value < 0.05 was deemed statistically significant. Besides, we carried out trial sequential analysis (TSA) using TSA Software (Copenhagen Trial Unit’s TSA Software®; Copenhagen, Denmark) to assess the risk of random errors [21].

## Results

### Eligible studies and the characteristics

We retrieved 103 records from PubMed, 295 records from Embase, 96 records from Cochrane Library and 28 records from CNKI. Briefly, the database search generated 522 articles, with 451 remaining after duplicate records were removed. Four hundred and thirty-four papers were later removed based on titles and abstracts because they were irrelevant to the meta-analysis. Nine of the 17 papers that underwent full-text review were further excluded for the following causes: 3 studies were performed not in surgical setting, 2 studies compared haloperidol with diazepam or ondansetron, 2 studies used the same data, and 2 studies were protocol. The steps of screening and choosing studies were presented in the flow diagram (Fig. 1). After reviewing the full text, we retained 8 suitable RCTs [17, 19, 22–27] with 1569 people [782 in the haloperidol groups and 787 in the control groups). All included studies were published from 1999 to 2021. Among them, 2 studies [26, 27] were from China, 3 studies [17, 22, 25] were from Japan, and the rest were performed in The United States [24], Netherlands [23] and Switzerland [19], respectively. Surgical types included orthopaedic surgery, thoracic surgery, gastrointestinal surgery, gynaecological surgery, cardiac surgery and vascular surgery. All included studies evaluated incidence of POD between haloperidol and control groups, ranging from 3 days to 7 days after surgery. Seven studies [17, 22–27] reported side effects of haloperidol (QTc interval prolongation, extrapyramidal symptoms and excessive sedation). There were four articles [19, 23, 26, 27] assessed the incidence of delirium by using Confusion Assessment Method of Intensive Care Unit (CAM-ICU) or Confusion Assessment Method (CAM). The rest used other diagnostic methods. The usage methods of haloperidol included PCIA [27], oral administration [23] and intravenous injection [17, 19, 22, 24–26]. The baseline characteristics of enrolled studies were shown in Table 1 (End of this manuscript).

**Table 1:** The basic characteristics of the enrolled studies

Author	Country	Age Cases/controls	Number Cases/controls	Surgical type	Administration Cases/controls
Kaneko (1999)	Japan	72.4/73.1	38/40	Gastrointestinal Surgery	5 mg daily for 5 days after surgery/ Saline
Kalisvaart (2005)	Netherlands	82.6/82.2	212/218	Hip-surgery	1.5 mg daily for 3 days after surgery/ Saline
Wang (2012)	China	74.0/74.7	229/228	Intra-abdominal Intra-thoracic Superficial Spinal and extremital	0.5 mg followed by continuous infusion of 0.1 mg hourly for 12 h/ Saline
Fukata (2014)	Japan	80.5/80.2	59/60	Abdominal and orthopedic surgery	2.5 mg daily for 3 days after surgery/ NA
Fukata (2016)	Japan	82.0/81.3	101/100	Abdominal and orthopedic surgery	5 mg daily for 3 days after surgery/ NA
Khban (2018)	America	60.0/62.3	68/67	Thoracic surgery	1.5 mg daily for 4 days after surgery/ Saline
Shao (2019)	China	70.6/71.3	30/30	Hip replacement surgery	PCIA:sufentanil 2μg•kg^-1^ + flurbiprofen 3 mg•kg^-1^ + haloperidol 5 mg/ PCIA:sufentanil 2μg•kg^-1^+ flurbiprofen 3 mg•kg^-1^
Hollinger (2021)	Switzerland	73.4/73.8	45/44	visceral, orthopaedic, vascular, gynaecological, cardiac, or thoracic surgery	5μg•kg^-1^ before the induction of anaesthesia/ Saline

Abbreviations: NA, not available

### Quality assessment and GRADE of evidence

**Fig. 2:**
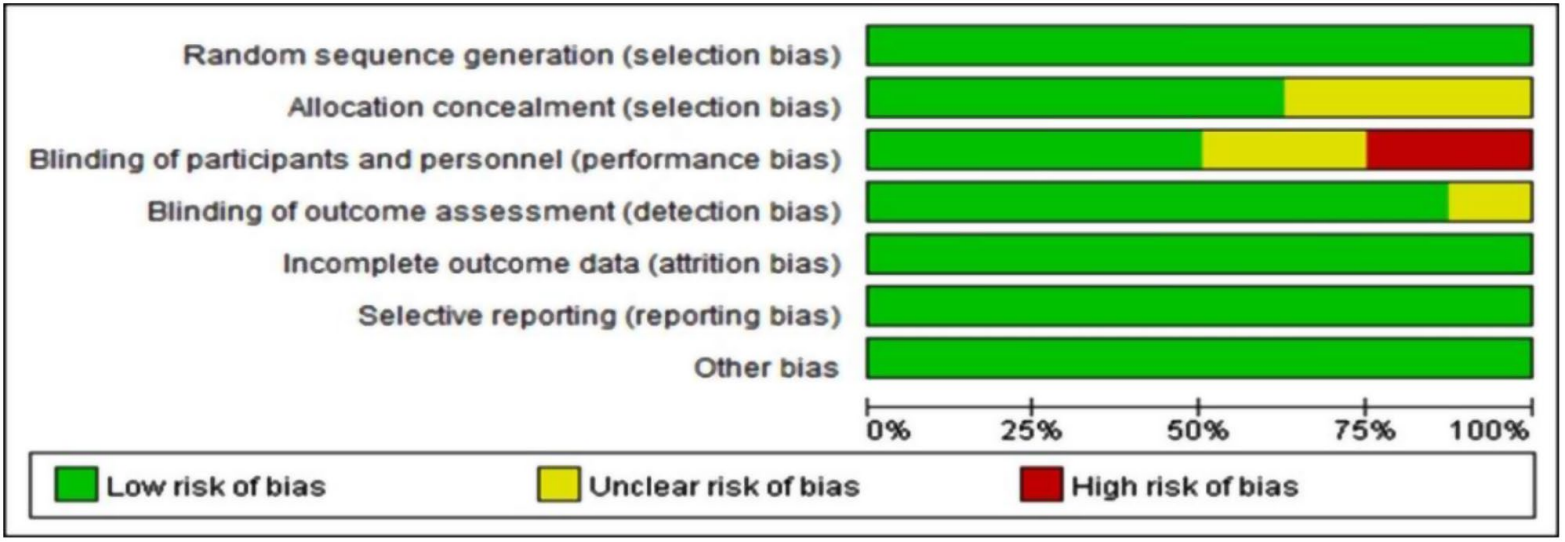
Risk of bias in the included studies

The most qualities of enrolled studies were categorized as ‘low risk’. All the enrolled studies introduced the random sequence generation method in detail. Four studies [19, 23, 24, 26] demonstrated double blinding, and the rest did not show who was blind to the allocation. The quality assessment and proportion of the risk of bias were exhibited in Fig. 2. What’s more, the GRADE assessment showed low and moderate levels of quality, which mainly attributed to the risk of bias and imprecise survey results (Supplementary Material 2).

### Effect of interventions

#### Postoperative delirium

**Fig. 3:**
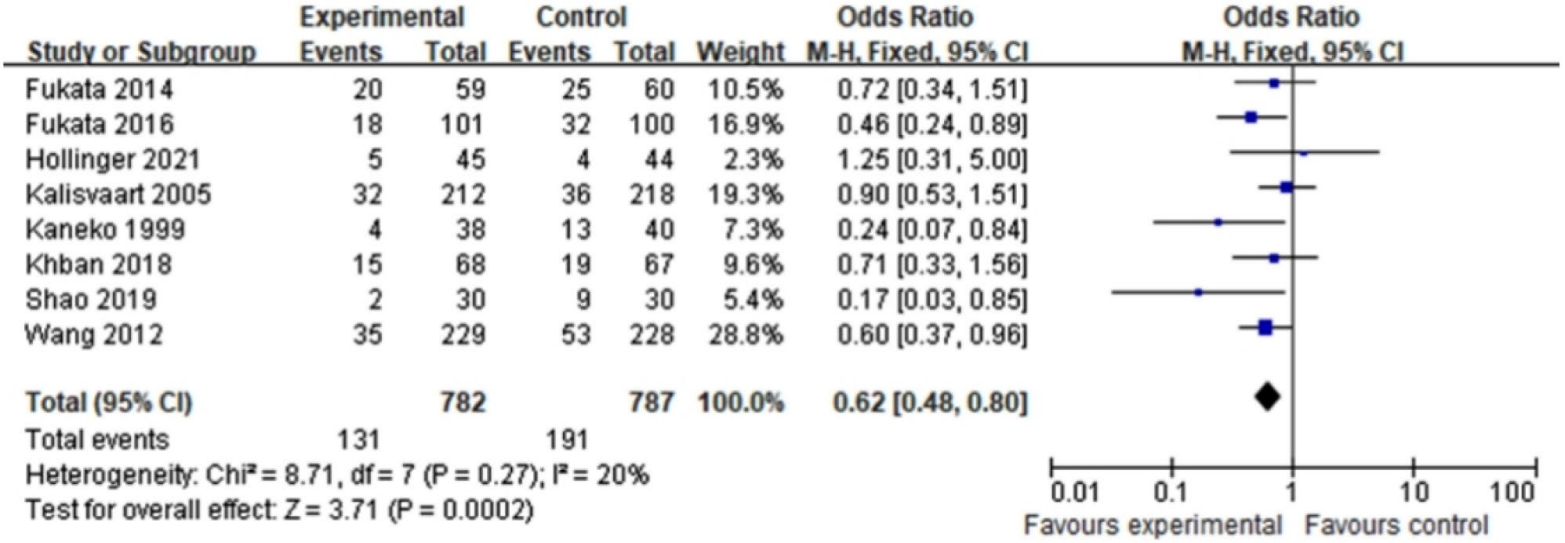
The effect of haloperidol versus control on POD incidence

**Fig. 4:**
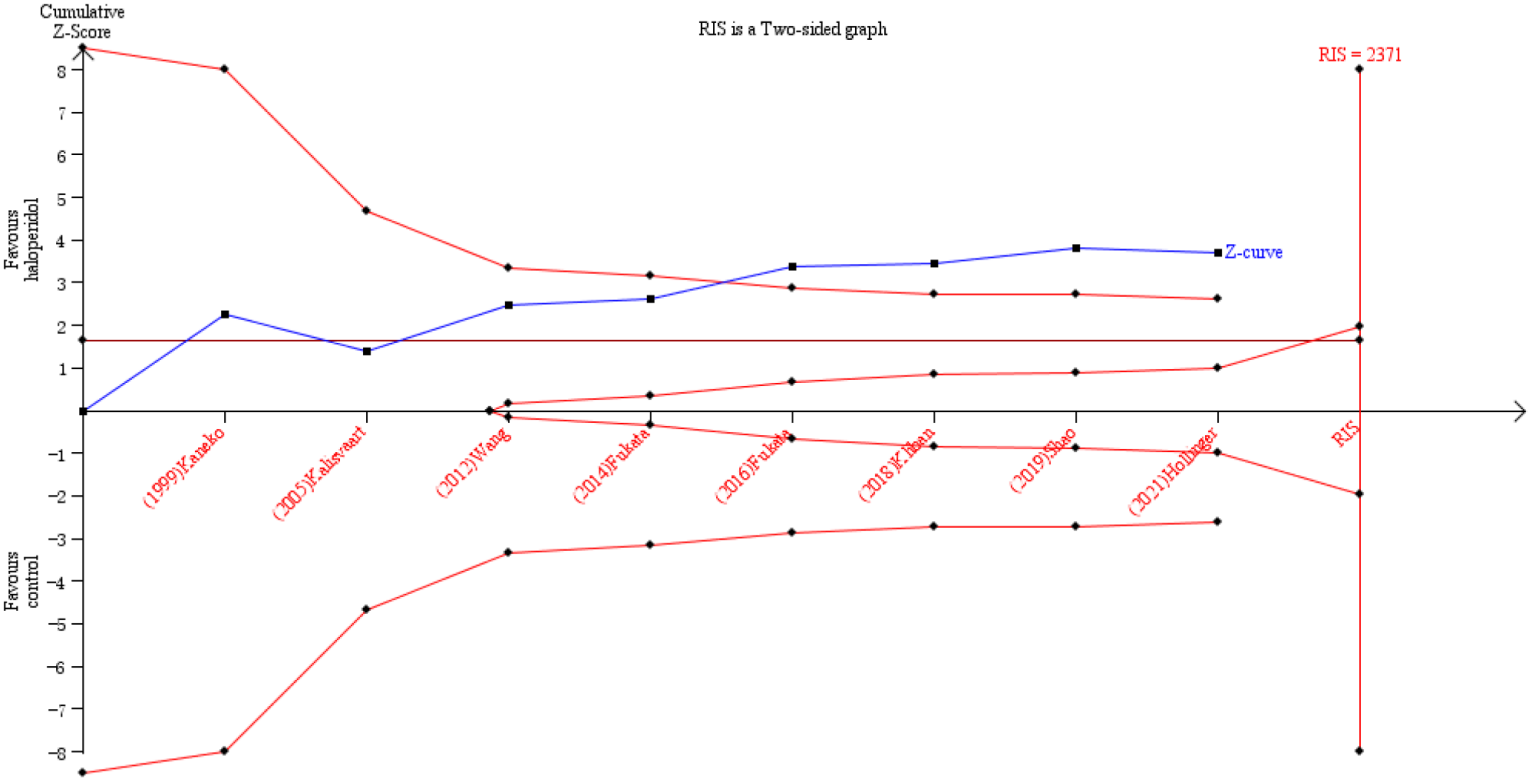
Trial sequential analysis for the effect of haloperidol versus control on POD incidence. RIS, required information size

**Fig. 5:**
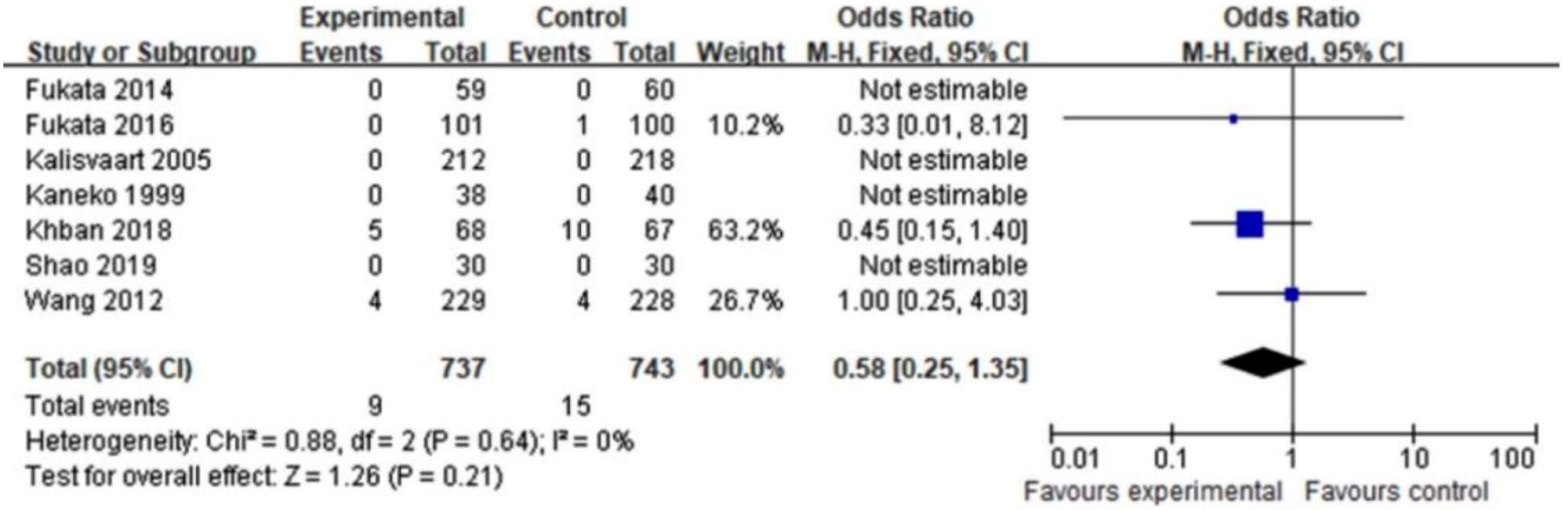
The effect of haloperidol versus control on haloperidol side effects

**Fig. 6:**
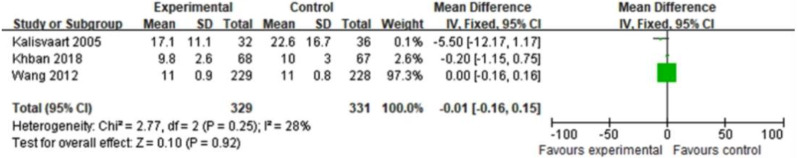
The effect of haloperidol versus control on the length of hospital stay

Given the fact that there was little heterogeneity among the enrolled studies, we utilized a fixed effects model to integrate the data concerning the incidence of POD. When data were combined, there was noticeable difference in the occurrence of POD between haloperidol and control groups (OR = 0.62, 95%CI 0.48–0.80, *P* = 0.0002, I^2^ = 20% Fig. 3). The cumulative Z-curve crossed the conventional and TSA-adjusted boundaries of benefit, showing haloperidol was beneficial in reducing POD (Fig. 4). Moreover, side effects of haloperidol and duration of hospitalization did not appear to be different between the experimental and the control groups (OR = 0.58, 95%CI 0.25–1.35, *P* = 0.21, I^2^ = 0%; MD =-0.01, 95%CI -0.16-0.15, *P* = 0.92, I^2^ = 28% Figs. 5 and 6). So haloperidol was a safe and efficient treatment for POD.

#### Subgroup analysis

**Fig. 7:**
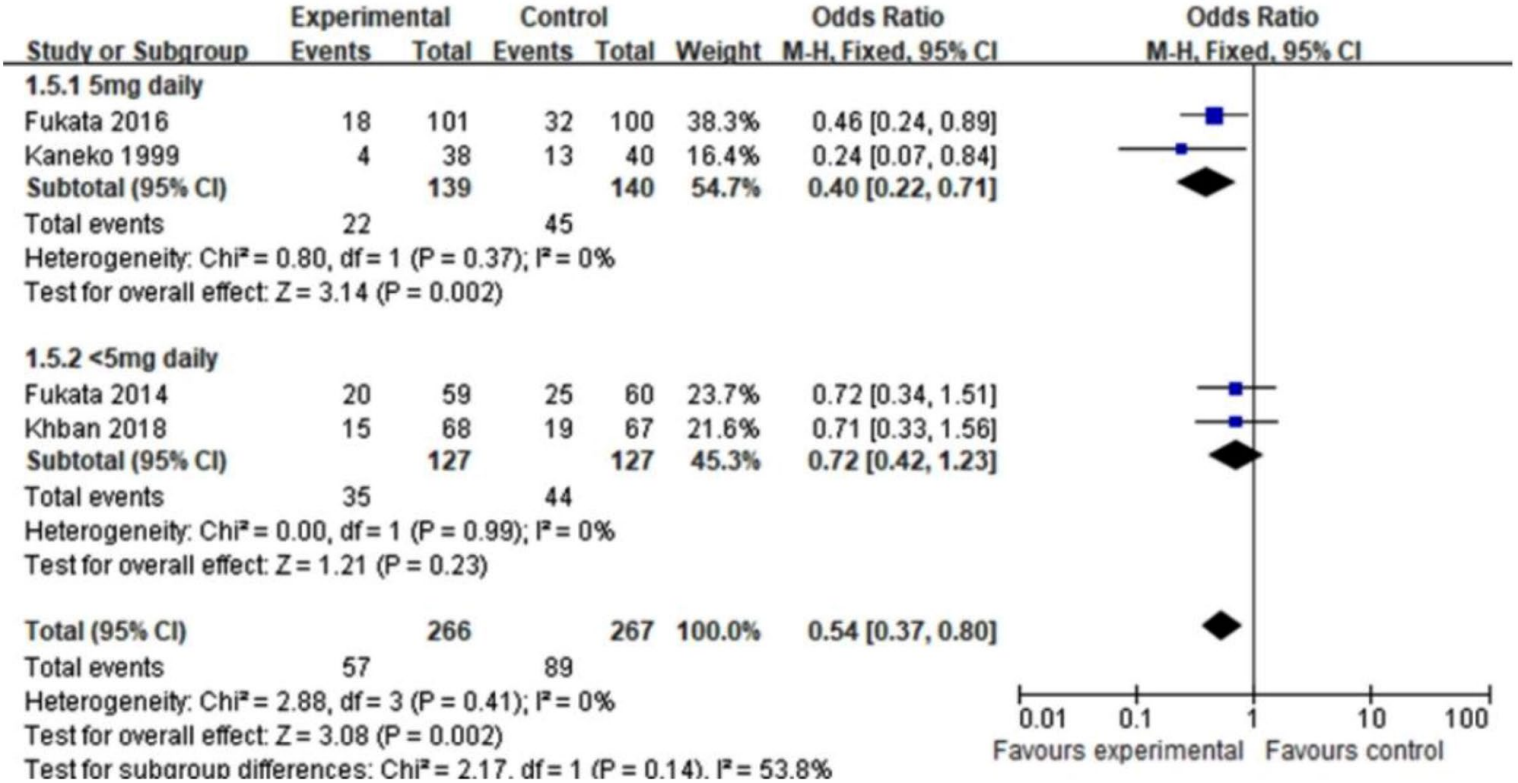
Subgroup analysis for POD incidence according to dose of haloperidol

Given the different dosages of haloperidol, we split the studies into two subgroups for analysis (5 mg daily and < 5 mg daily). Interestingly, high-dose haloperidol (5 mg daily) was able to improve POD, whereas low-dose haloperidol (< 5 mg daily) did not (5 mg daily: OR = 0.40, 95%CI 0.22–0.71, *P* = 0.002, I^2^ = 0%; <5 mg daily: OR = 0.72, 95%CI 0.42–1.23, *P* = 0.23, I^2^ = 0% Fig. 7), which suggested that the high-dose haloperidol (5 mg daily) possessed a promising potential in reducing the POD.

### Sensitivity analysis and publication bias

**Fig. 8:**
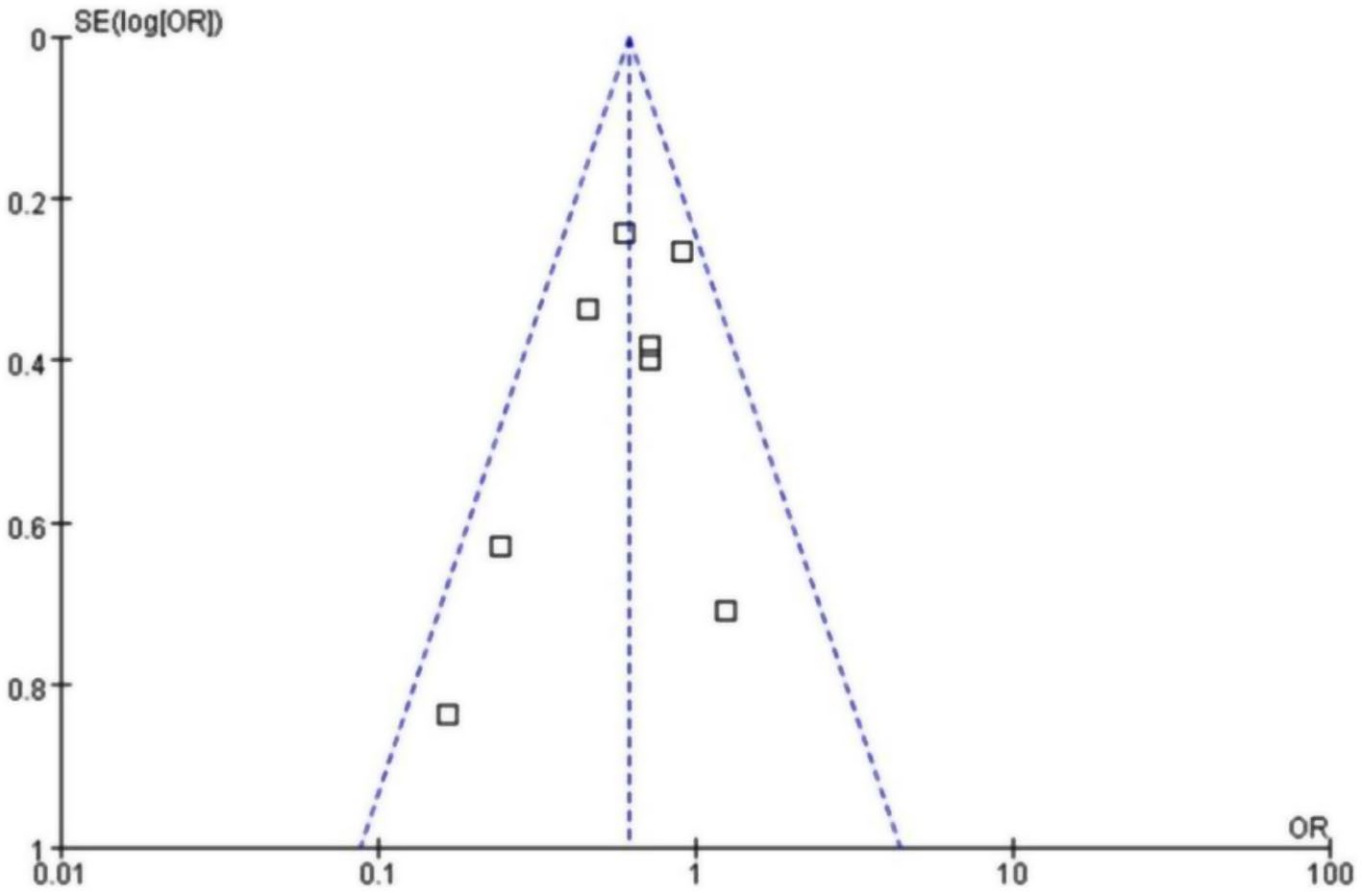
The funnel plot

The sensitivity analysis of primary outcomes was conducted by eliminating individual study each time. As a result, pooled data were still statistically significant, indicating its stability and reliability (Table 2). Moreover, no obvious publication bias was discovered through examining the funnel plots (Fig. 8).

**Table 2:** The sensitivity analysis of haloperidol’s effect on POD incidence

Study excluded	OR (95% CI)	I^2^ (%)	P for Cochrane’s Q test	P for overall effect
Fukata (2014)	0.16 [0.47,0.80]	30	0.20	0.0003
Fukata (2016)	0.65 [0.50,0.86]	22	0.26	0.002
Hollinger (2021)	0.61 [0.47,0.78]	23	0.26	0.0001
Kalisvaart (2005)	0.56 [0.42,0.74]	6	0.38	0.0001
Kaneko (1999)	0.65 [0.50,0.84]	5	0.39	0.001
Khban (2018)	0.61 [0.47,0.80]	30	0.20	0.0003
Shao (2019)	0.65 [0.50,0.84]	1	0.41	0.0009
Wang (2012)	0.63 [0.47,0.85]	31	0.19	0.002

Abbreviations: OR: Odds ratios; I^2^: *I*-square

## Discussion

The current meta-analysis revealed that administration of haloperidol could reduce POD occurrence in elderly patients. Furthermore, there was no apparent side effects and the application of haloperidol did not affect the patient’s hospitalization time. In a word, all findings supported the idea that haloperidol should be considered a prophylactic treatment against POD in elderly patients.

POD occurred in the hospital up to seven days after surgery or until discharge [28], which was described by short-term fluctuations in mental status, attention and level of awareness [29]. It usually happened in the operating room or PACU at any point during or shortly after emergence from general anaesthesia. According to clinical manifestations, delirium was classified as hypoactive (decreased alertness, motor activity and anhedonia), hyperactive (agitated and combative) and mixed forms [30]. Notably, hypoactive delirium was common in elderly patients because its clinical manifestations were relatively hidden [31]. Moreover, POD was connected to a number of negative outcomes, including cognitive dysfunction, extended hospital stays, increased mortality and higher healthcare expenses [32]. Therefore, it was urgent and essential to adopt effective management strategies to improve POD.

In clinical practice, multiple medications were utilized to treat POD, and haloperidol was one of the most common [33]. The European Society of Anaesthesiology also recommended haloperidol for the treatment of POD in small doses orally or intramuscularly [34], even though the fact that haloperidol administration carried dangers like extrapyramidal responses, arrhythmias, and cardiac damage [35]. Moreover, haloperidol should be intravenously administered slowly under the condition of monitoring the electrocardiogram [34]. A study reported intravenously administration of haloperidol at the beginning of delirium considerably improved POD in elderly patients [17]. Recently, Hollinger discovered that the use of haloperidol could not improve POD [19]. In view of the controversy, we conducted the meta-analysis to systematically examine the effectiveness of haloperidol in POD.

Until now, the concrete pathophysiology of POD was still ambiguous. The majority of individuals agreed that predisposing factors and precipitating factors worked together to trigger POD [5]. Early recognition of risk factors was believed to be an effective approach to reduce POD [36]. Risk factors included advanced age, comorbidities, preoperative fluid fasting, type of surgery (abdominal and cardiothoracic), intraoperative bleeding, prolonged time of surgery, bispectral index (too low or too high), intraoperative electrolyte disturbance and postoperative pain [5]. Oxidative stress, brain structure or function damage, neurotransmitter imbalance and thermoregulation disorder were some potential causes of POD [37]. Many hypotheses suggested that the underlying causes of POD included dopamine activity and cholinergic deficiency [38]. In reality, dopamine inhibited acetylcholine release by activating the dopamine receptor, whereas blocking the receptor could increase acetylcholine release [39]. Fortunately, haloperidol, a butyryl benzene antipsychotic, could effectively regulate the balance of dopamine and acetylcholine in the brain by blocking the dopamine D2 receptor, which alleviated impairment in memory function and spatial cognition and alleviated POD by modulating neurotransmitter balance [40]. Hence, it was reasonable to hypothesize that haloperidol might be useful in preventing POD.

In this research, haloperidol did decrease the occurrence of POD in elderly patients, which was similar to previous studies [41–43], in which haloperidol administration during the perioperative phase was linked to significant decreases in POD incidence and symptom relief. Nevertheless, another study indicated that haloperidol did not improve occurrence of delirium in adult hospitalized ICU patients, which might be attributed to differences in the participant population [44]. Moreover, a meta-analysis revealed that haloperidol did not drastically reduce delirium occurrence in ICU patients, while it did lower POD solely [45]. Although GRADE assessment showed low quality, TSA confirmed the accuracy of this result and the robustness was demonstrated further by sensitivity analysis. Given the above, we might speculate haloperidol could decrease the occurrence of delirium, at least in postoperative patients.

Meanwhile, there was no difference in the side effects of haloperidol, which might be explained by the fact that side effects of haloperidol often occurred in patients with long-term high-dose use of haloperidol [27]. Additionally, children and adolescents were at high risk of side effects from haloperidol [46]. It reported that a low amount of haloperidol (1 mg twice daily) as a preventive measure did not lower the number of cases of delirium [47], which was in line with the outcome of this meta-analysis that high-dose haloperidol (5 mg daily) could improve POD while the low-dose haloperidol (< 5 mg daily) could not. Similar to this meta-analysis, Shen et al. found a dose of 5 mg haloperidol daily could help ameliorate POD [43].

This was the first meta-analysis to examine the role of haloperidol’s perioperative application on POD without any restrictions concerning haloperidol’s doses or type of surgery in elderly patients. However, there were certain restrictions in our research. Firstly, the analysis of outcomes used a small sample size due to the few included research, which may lead to biased results. Secondly, the inclusion and exclusion criteria, Body Mass Index, outcome measures, duration of operation, and surgical blood loss, differed among the recruited studies, which might contribute to heterogeneity. Lastly, we failed to assess the long-term side effects of haloperidol due to limited data. To further evaluate the efficacy of haloperidol on POD, large and properly designed randomized trials are urgently required.

## Conclusion

In general, the meta-analysis comprehensively and systematically analysed all included articles. Statistical data from research demonstrated that haloperidol administration authentically decreased the incidence of POD without evident side effects among old patients and high-dose haloperidol (5 mg daily) was a promising alternative.

### Electronic supplementary material

Below is the link to the electronic supplementary material.

**Supplementary Material 1:** PRISMA checklist

## Data Availability

The datasets used and/or analysed during the current study are available from the corresponding author on reasonable request.

## Abbreviations

POD: Postoperative delirium
CNKI: China National Knowledge Infrastructure
MeSH: Medical subject headings
MD: Mean difference
CI: Confidence interval
OR: Odds ratio
CAM-ICU: Confusion Assessment Method of Intensive Care Unit
CAM: Confusion Assessment Method
RIS: Required information size
